# Spousal Care and Pain Among the Population Aged 65 Years and Older: A European Analysis

**DOI:** 10.3389/fmed.2021.602276

**Published:** 2021-05-11

**Authors:** Fátima Barbosa, Alice Delerue Matos, Gina Voss, Patrício Costa

**Affiliations:** ^1^Communication and Society Research Centre, Institute of Social Sciences, University of Minho, Braga, Portugal; ^2^Department of Sociology, Institute of Social Sciences, University of Minho, Braga, Portugal; ^3^School of Medicine, Life and Health Sciences Research Institute, University of Minho, Braga, Portugal; ^4^ICVS (Life and Health Sciences Research Institute)/3B's (Biomaterials, Biodegradables and Biomimetics) Associate Laboratory, Guimarães, Portugal; ^5^Faculty of Psychology and Education Sciences, University of Porto, Porto, Portugal

**Keywords:** spouse caregivers, 65 plus, reported pain, Europe, SHARE

## Abstract

**Background:** Spousal care is the most important source of informal care in old age. Nevertheless, despite the growing importance of this issue, the association between providing spousal care inside the household and pain remains unexplored in Europe.

**Objective and Methods:** This study aims to estimate the prevalence of pain reported by spouse caregivers aged 65 plus that provide care inside the household and to investigate the association between providing spousal care and pain. Data from 17 European countries that participated in wave 6 of the Survey of Health, Aging and Retirement in Europe (SHARE) is used. The analyses are based on 26,301 respondents aged 65 years and older who provide informal care inside the household to their spouse/partner exclusively (*N* = 1,895) or do not provide any informal care (inside or outside the household) (24,406). Descriptive statistics and multilevel logistic regressions (individual-level as level 1, and country as level 2) were performed.

**Results:** Overall, spouse caregivers report pain more often (63.4%) than their non-caregiver‘s counterparts (50.3%). Important differences in the prevalence of pain among spouse caregivers were found between countries, with Portugal (80.3%), Spain (74.6%), France (73%), Italy (72.4%), and Slovenia (72.1) showing the highest prevalence of pain, and Denmark (36%), Switzerland (41.5) and Sweden (42.3%), the lowest. Results from multilevel logistic regressions show that European individuals aged 65+ who provide spousal care have an increased likelihood of reporting pain (OR 1.30; CI = 1.13–1.48).

**Conclusion:** Our results suggest that in Europe, spouse caregivers aged 65+ are at greater risk of experiencing pain. Therefore, European policymakers should consider spouse caregivers as a health priority group, and take measures to ensure they receive comprehensive health and socio-economic support.

## Introduction

In old age, spouse caregivers are considered to be the most important source of informal care (1). Studies indicate that spouse caregivers are particularly vulnerable, as they are older and report worse physical and mental health than the non-caregivers (2, 3). Spouse caregivers usually live with the care recipient, provide more hours of care, and find less respite from their caregiver role than other caregivers (4). The majority report having no choice in taking up the caregiving role (5), they provide higher levels of care (6), and many are solo caregivers (7). Compared to other caregivers, spouse caregivers were found to develop arthritis and chronic back pain several years after the initial caregiving experience (8). Among informal caregivers, pain is also significantly associated with the caregiver burden (9, 10) and with an overestimation of the pain of the cared-for person (11).

In Europe, as well as the rest of the world, pain is considered to be a major health problem (12–15). Globally, pain and pain-related diseases are associated with years of life with disability and disease burden (16–18). According to Dorner (19), chronic pain is determined by biological, psychological, and social aspects and is linked with mental disorders, sleep disturbances, and demographic and socioeconomic factors. Pain has a higher prevalence in women (13, 14, 20, 21), in people with lower socioeconomic status (13), and in older ages (21–24). The prevalence of pain in people aged over 60 is twice that in younger people (23), with recent studies showing that 45–85% of older people experience pain (23). Additionally, older adults are at a greater risk of having their pain managed and treated inadequately (12, 25), which has a significant impact on an individual and social level.

At the individual level, chronic pain is associated with an increased incidence of adverse outcomes, such as functional impairment, disability, falls, depression/ anxiety disorders, sleep disturbances, obesity, frailty, social isolation, and death (14, 20, 22, 26–30). Chronic pain is also associated with a high personal burden (31), with a significant impact on the quality of life and the performance of everyday activities (32–34). At the social level, chronic pain implies a higher demand for healthcare which can lead to an economic burden (22, 35), which impacts public health systems (15, 31, 35, 36).

Literature shows that there is considerable variation in pain across European countries (13, 20). Cimas et al. (20) found that in Europe, for older adults, the prevalence of pain is higher in Southern Mediterranean countries (Italy, France, and Spain) and Eastern European countries (Estonia and Slovenia) and lower in the Northern countries (Denmark, Netherlands, and Sweden) and Switzerland.

Taking into consideration the fact that spouses, regardless of their gender, socioeconomic background, and welfare policy contexts, are an essential source of informal care in old age (1) and that informal care provided by spouses or partners is projected to increase in the coming years (37), it is crucial to analyse the association between providing spousal care and pain. Furthermore, considering that spousal care is more prevalent among older individuals (4, 38), this research aims to examine whether providing spousal care inside the household at older ages (65+) in Europe is associated with pain. More precisely, the main objectives of this article are as follows: (1) to estimate the prevalence of pain reported by spouse caregivers aged 65+ and (2) to investigate the association between providing spousal care and pain.

The results of this investigation will provide vital information for researchers and policymakers in order to improve the health and well-being of European spouse caregivers that provide care inside the household.

## Materials and Methods

### Data Source

This work uses the Survey of Health, Ageing and Retirement in Europe (SHARE) data, wave 6 (2015), release 7.0.0. (10.6103/SHARE.w6.700) (39). SHARE is a European multidisciplinary and cross-national panel database of microdata on health, socioeconomic status, and social and family networks (40). The SHARE target population consists of everyone aged 50 years and over at the time of sampling (probability sampling) who have their regular domicile in the respective SHARE country. The partner of the sampling respondent is eligible for an interview as well regardless of age. The interviewers used computer-assisted personal interviewing (CAPI) to collect the data. Proxy interviews were allowed when respondents were unable to do an interview, for example, for health reasons. For further methodological details of the SHARE project, please see Börsch-Supan et al. (40).

The SHARE study is guided by international research ethics principles, such as the Respect Code of Practice for Socio-Economic Research and the “Declaration of Helsinki.” SHARE wave 6 was reviewed and approved by the Ethics Council of the Max Planck Society.

### Study Sample

SHARE wave 6 covers 17 European countries (Austria, Germany, Sweden, Spain, Italy, France, Denmark, Greece, Switzerland, Belgium, Czech Republic, Poland, Luxembourg, Portugal, Slovenia, Estonia, and Croatia) plus Israel. In wave 6, a total of 68,188 interviews were conducted with individuals aged 50 plus. For this study, as a first step, we restricted our sample to SHARE European respondents aged 65+ who answered the informal co-residential question (*N* = 27,939). This means that only people living with one or more persons were included in our analysis. As a second step, in order to compare spouse caregivers who provide informal care inside the household with non-caregivers, we excluded individuals who provide care inside the household to persons other than a spouse, and also individuals who provide informal care outside the household (*N* = 1,638). Therefore, a total of 26,301 individuals were considered in our analysis: Austria (1,376), Germany (1,675), Sweden (1,969), Spain (2,802), Italy (2,231), France (1,357), Denmark (1,239), Greece (1,917), Switzerland (1,181), Belgium (1,832), Czech Republic (1,947), Poland (714), Luxembourg (504), Portugal (779), Slovenia (1,698), Estonia (2,208), and Croatia (872).

### Measures

#### Outcomes

In wave 6, participants were asked whether they were “troubled by pain” (ph084_). Those who answered “Yes” were categorized as 1 and those who answered “No” as 0.

#### Independent Variable

The provision of informal spouse care inside the household was assessed using the following question: “Is there someone living in this household whom you have helped regularly during the last 12 months with personal care, such as washing, getting out of bed, or dressing? By regularly, we mean daily or almost daily during at least 3 months.” If the respondent answered affirmatively, the following question was asked: “To whom do you give help in this household?”

To avoid problems of misclassification, individuals who provide care inside the household to persons other than a spouse were excluded. Moreover, individuals who provide informal care outside the household (such as dressing, bathing or showering, helping other(s) with eating, getting in or out of bed, or using the toilet) and who answered “Yes” to the question “In the last 12 months, have you personally given any kind of help listed on this card to a family member from outside the household, a friend, or neighbor?” were also excluded from this study.

Consequently, ~6% of European respondents aged 65+ who answered the informal co-residential question were excluded. Therefore, spouse caregivers who provide care to a spouse and no one else inside the household were coded as 1, and non-caregivers were coded as 0.

#### Confounders

Based on the literature review, we identified important confounders to be included in our analysis. Age of respondent at the time of interview and gender (1 female and 0 male). Education was measured according to the highest level of education attained using the standardized coding of the International Standard Classification of Education (ISCED-97). We divided the ISCED-97 codes into three groups: **low level of education**, which included ISCED-97 codes 0 (no education), 1 (primary education), and 2 (lower secondary education); **medium level of education**, which included codes 3 (secondary) and 4 (post-secondary non-tertiary education); and **high level of education**, which included participants with ISCED-97 codes 5 (first stage of tertiary education) and 6 (second stage of tertiary education).

Income was constructed using the variable total household net income (version A) that is obtained by a suitable aggregation at the household level of all individual income components. Income was adjusted for purchasing power parity and the square root of household size and divided into tertiles. The lowest tertile was coded as 1, the middle as 2, and the highest as 3.

The number of limitations in instrumental activities of daily living (IADL) includes the assessment of nine instrumental activities (using a map, preparing a hot meal, shopping, using a telephone, taking medication, doing housework or gardening, managing money, leaving the house independently and accessing transportation services, and doing personal laundry) for a period of more than 3 months. The number of limitations in activities of daily living (ADL) was assessed according to the presence of difficulties with dressing, walking, bathing, eating, getting in or out of bed, and using the toilet for a period of more than 3 months. The presence of chronic diseases was measured based on the multiple answer question “*Has a doctor ever told you that you had* …” that asks which of the listed chronic conditions the respondents had according to their doctors.

Physical inactivity was assessed using the generated dummy variable *phactiv*. This variable is constructed based on SHARE questions: br015_, which is related to the frequency of vigorous activity (i.e., sport, heavy housework, or a job that requires physical labor), and br016_, which is related to the frequency of moderate physical activity (i.e., activities requiring a low or moderate level of energy, such as gardening, cleaning the car, or walking). Both questions have four response options: (1) more than once a week; (2) once a week; (3) one to three times a month; and (4) hardly ever or never. Physically inactive individuals are those who have hardly ever or never practiced vigorous or moderate physical activity.

Depression symptoms (EURO-D Caseness) was measured by having four or more symptoms in the EURO-D 12-item scale (feelings of depression, pessimism, wishing death, guilt, irritability, tearfulness, fatigue, sleeping troubles, loss of interest, loss of appetite, reduction in concentration, and loss of enjoyment) over the last month (41). The presence of affective or emotional disorders was assessed by question ph006d18 (*Has a doctor ever told you that you had/Do you currently have other affective or emotional disorders, including anxiety, nervous, or psychiatric problems?*).

Lastly, satisfaction with life is measured using question AC012_ (*On a scale from 0 to 10 where 0 means completely dissatisfied and 10 means completely satisfied, how satisfied are you with your life?*). This variable ranges from 0 to 10, with the highest scores meaning higher life satisfaction.

### Statistical Analysis

**Figure 1:**
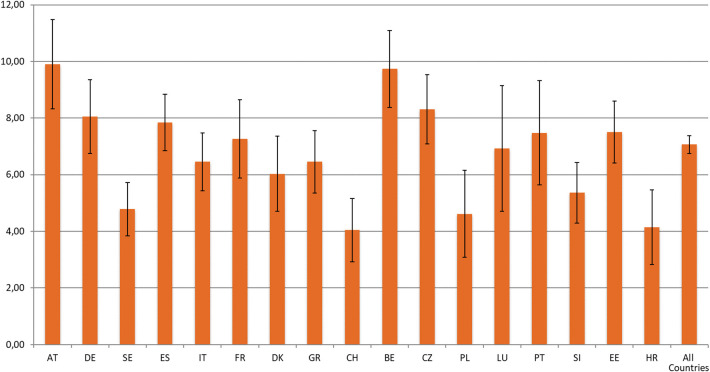
Prevalence of spouse caregiving provided inside the household by individuals aged 65+, according to country. Source: SHARE, release 7.0.0., wave 6, weighted data, *N* = 26,301. Brackets denote 95% confidence intervals. Countries: Austria (AT); Germany (DE); Sweden (SE); Spain (ES); Italy (IT); France (FR); Denmark (DK); Greece (GR); Switzerland (CH); Belgium (BE); Czech Republic (CZ); Poland (PL); Luxembourg (LU); Portugal (PT); Slovenia (SI); Estonia (EE); Croatia (HR).

**Figure 2:**
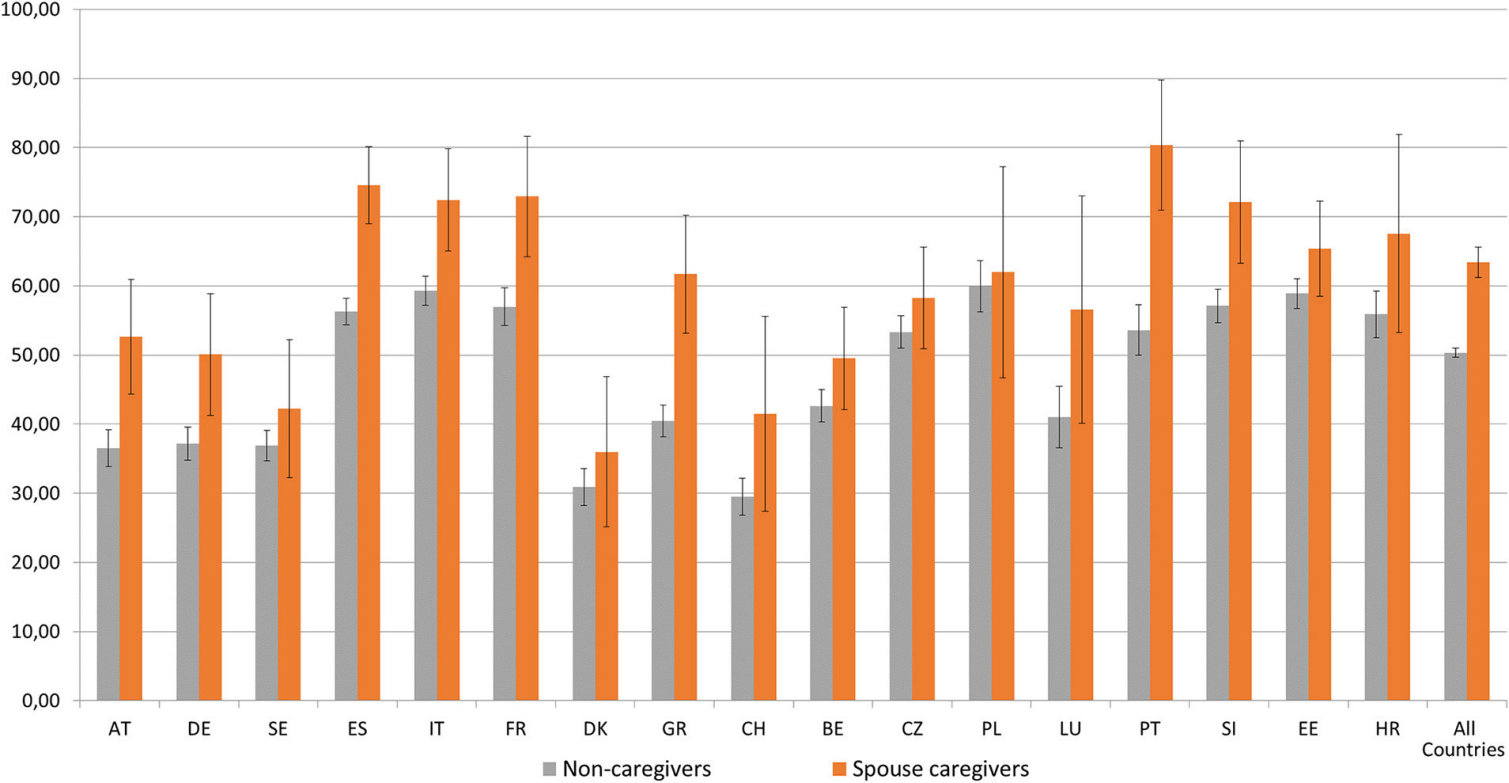
Prevalence of self-reported pain by people aged 65+ by country and caregiver status. Source: SHARE, release 7.0.0., wave 6, weighted data, *N* = 26,301. Brackets denote 95% confidence intervals. Countries: Austria (AT); Germany (DE); Sweden (SE); Spain (ES); Italy (IT); France (FR); Denmark (DK); Greece (GR); Switzerland (CH); Belgium (BE); Czech Republic (CZ); Poland (PL); Luxembourg (LU); Portugal (PT); Slovenia (SI); Estonia (EE); Croatia (HR).

Firstly, a missing data analysis was performed. We found missing values higher than 5% in the economic and health variables and therefore used SHARE multiple imputations to maximize the number of observations (42). After including these imputed variables, the missing data were residual (lower than 1%). Moreover, since the sample design is not uniform between the different countries, calibrated individual weights were used in all the descriptive statistical analyses (Figures 1, 2 and in the percentages in Table 1).

**Table 1:** Descriptive statistics of European older adults aged 65+, according to caregiver status.

	***N***	**Non-caregivers**	**Spouse caregivers**	**T/χ^2^**	***p*-value**	**Cohen's d/phi**	**CI**
		***N* = 24,406**	***N* = 1,895**				
Age, years mean (SD)	26, 301	73.99 (6.89)	75.99 (6.84)	−9.953	0.000	−0.237^*^	−0.284 to −0.191
Female (%)	26, 301	48.88	53.48	24.763	0.000	0.031	0.019 to 0.043
Education (%)	26, 301			47.673	0.000	0.043	0.031 to 0.055
Low		51.6	57.19				
Medium		29.36	29.1				
High		19.04	13.71				
Income (%)	26, 299			36.888	0.000	0.037	0.025 to 0.050
Lowest tertile		34.86	34.07				
Meddle tertile		36.79	39.9				
Highest tertile		28.34	26.04				
IADL, mean (SD)	26, 301	0.83 (2.05)	1.10 (1.99)	−6.211	0.000	−0.148	−0.195 to −0.101
ADL, mean (SD)	26, 301	0.40 (1.18)	0.58 (1.24)	−6.334	0.000	−0.151	−0.198 to −0.104
Chronic diseases, mean (SD)	26, 301	1.46 (1.29)	1.68 (1.37)	−7.450	0.000	−0.178	−0.224 to −0.131
Physical inactivity (%)	26, 301	19.16	24.71	27.295	0.000	0.032	0.020 to 0.044
Depression (4 or more depressive symptoms) (%)	26, 301	30.33	48.27	234.312	0.000	0.094	0.082 to 0.106
Affective or emotional disorders (%)	26, 286	7.19	9.36	32.859	0.000	0.035	0.023 to 0.047
Life satisfaction, mean (SD)	26, 301	7.71 (1.75)	7.17 (1.94)	11.441	0.000	0.273^*^	0.226 to 0.320
Troubled with pain (%)	26, 288	50.33	63.42	114.689	0.000	0.066	0.054 to 0.078

*Source: SHARE, release 7.0.0., wave 6, weighted data, N = 26,301*.

*Tests for effect size: Cohen's d:*

* small effect (≥0.20); ^**^medium effect (≥0.50); ^***^large effect (≥0.80); Phi: ^*^small effect (≥0.10); ^**^medium effect (≥0.30); ***large effect (≥0.50).

Secondly, the prevalence of spousal care provided by individuals aged 65+ was assessed by country. Thirdly, the prevalence of self-reported pain among people aged 65+ was estimated by country and caregiver status. Fourthly, a descriptive and bivariate analysis was applied to analyse differences between the non-caregiver group and the spouse caregiver group. For categorical data frequencies with percentages and chi-square tests were performed. For continuous data, the mean with standard deviation and independent *t*-test were applied. Effect size measures (Cohen's d/Phi) with a 95% confidence interval were used to complement the analysis. Lastly, multilevel logistic regressions with individual-level as level 1 and country as level 2 were performed to investigate the association between providing spousal care inside the household and pain. In the first phase, a null model (Model 0) was calculated to analyse the variance of pain due to country differences. Considering that the intra-class correlation coefficient (ICC) of the null model was 5.5%—higher than the recommended cut-point of 5% for using Multilevel Modeling (43, 44)—we moved to Model 1. In this model, the individual level indicators were introduced (age, gender, education, income, IADL, ADL, chronic diseases, physical inactivity, depression, affective emotional disorders, and life satisfaction). In addition, continuous indicators were centered. In Model 2, the spousal care indicator was introduced. Odds ratios (OR), 95% confidence intervals (IC), *p*-value (*p*), intra-class correlation coefficient (ICCcountry), between-country variance, and deviance of the statistical model are presented. Additionally, classical standard errors and robust standard errors were applied. Since no differences were found, classical standard errors are presented in this analysis (45). Due to singularity fit problems, country cross-level interactions were not considered in this research.

For statistical analysis, the IBM SPSS 25 (46) and software R 4.0.2. (47) were used.

Descriptive and bivariate analyses were executed in IBM SPSS. Multilevel logistic regression analyses were performed in software R using the function “glmer” of the lme4 package (48). Function “icc” of the package “performance” was also used to calculate the intraclass-correlation coefficient (ICC) for mixed-effects models (49).

## Results

The percentage of individuals aged 65+ who provide informal spouse care inside the household, by country, is shown in Figure 1. Overall, the prevalence of spousal care provided inside the household by individuals aged 65+ in Europe is 7.1%. The highest prevalence of spousal care is found in Austria (9.9%), Belgium (9.7%), and the Czech Republic (8.3%). By contrast, Switzerland (4%), Croatia (4.2%), and Poland (4.6%) are the countries with the lowest percentage of this type of informal care.

Figure 2 shows the prevalence of self-reported pain by people aged 65+ by country and caregiver status. The data highlight differences in self-reported pain between non-caregivers and spouse caregivers and within the analyzed countries. Overall, spouse caregivers report pain more often (63.4%) than their non-caregiver counterparts (50.3%).

In eight countries (Austria, Germany, Spain, Italy, France, Greece, Portugal, and Slovenia) spouse caregivers, compared to their non-caregiver counterparts, reported significantly higher percentages of pain. Portugal (80.3%), Spain (74.6%), France (73%), Italy (72.4%), and Slovenia (72.1) are the countries with the highest prevalence of pain among spouse caregivers. By contrast, in Sweden, Denmark, Switzerland, Belgium, the Czech Republic, Poland, Luxembourg, Estonia, and Croatia, no significant differences were found between groups. Moreover, Denmark (36%), Switzerland (41.5), and Sweden (42.3%) are the countries with the lowest percentage of spouse caregivers reporting pain.

Table 1 shows the descriptive analysis (number, frequencies or mean, and standard deviation) and bivariate (*t*-test and chi-square test) analysis, as well as the effect size measures of Europeans aged 65+, according to caregiver status. The data show that the group of spouse caregivers are older and predominantly women in comparison with the non-caregiver group. Regarding education and income, the group of spouse caregivers, compared to their non-caregiver counterparts, shows higher percentages of lower educational levels and higher percentages of individuals with middle income, and lower percentages of individuals with high income. Concerning physical health, the spouse caregiver group reports a higher number of IADL and ADL limitations, as well as a higher number of chronic diseases and higher levels of physical inactivity. Higher percentages of four or more depressive symptoms and affective or emotional disorders were also found in the spouse caregiver group compared to the non-caregiver group. The group of spouse caregivers showed lower levels of satisfaction with life compared with the non-caregiver group. Lastly, the spouse caregiver group reported pain more often compared with the group of non-caregivers. However, when considering the effect size, which measures the magnitude of the differences found, these differences are only significant for age and life satisfaction (Cohen's d: small effect ≥0.20 but <0.50). The group of spouse caregivers is significantly older and less satisfied with life compared with the non-caregiver group.

Table 2 presents the results of the multilevel logistic regression analysis. Model 0 (null model) shows that differences between countries can explain 5.5% (ICC 0.055) of the variation in pain. In Model 1, the individual-level variables were considered. The results from Model 1 show that, with exception of IADL, all the other characteristics of the individuals are associated with pain. The data indicate that, with advanced age, people have a higher likelihood of reporting pain (OR = 1.06; CI = 1.02–1.09). Women (OR = 1.74; CI = 1.65–1.84) and people with a higher number of ADL limitations (OR = 1.28; CI = 1.22–1.34) and a higher number of chronic diseases (OR = 1.38; CI = 1.35–1.42) also show a higher likelihood of reporting pain. Conversely, people with medium (OR = 0.84; CI = 0.78–0.90) and high (OR = 0.80; CI = 0.74–0.86) educational levels and higher income (OR = 0.92; CI = 0.86–0.99) show a lower likelihood of reporting pain. Additionally, people who reported four or more depressive symptoms (OR = 1.86; CI = 1.75–1.99), affective or emotional disorders (OR = 1.52; CI = 1.34–1.72), and are physically inactive (OR = 1.26; CI = 1.15–1.38) show a higher likelihood of reporting pain. Lastly, people with higher levels of life satisfaction show a decreased likelihood of reporting pain (OR = 0.84; CI = 0.82–0.87). Model 1 also illustrates how differences between countries can explain about 3% (ICC 0.030) of the variation in reported pain. Model 2 shows that, after including the spousal care variable, all variables become stable. In this model, people who provide spousal care inside the household have an increased likelihood of reporting pain (OR = 1.30; IC = 1.13–1.48). When this variable is included, Model 2 shows that about 3% (ICC 0.030) of the variation in reported pain can be explained by differences between countries.

**Table 2:** Multilevel logistic regression with troubled with pain as the outcome.

	**Model 0**			**Model 1**			**Model 2**		
	**OR**	**CI (95%)**	***p***	**OR**	**CI (95%)**	***p***	**OR**	**CI (95%)**	***p***
**Fixed parts**									
(Intercept)	0.92	0.75–1.14	0.462	0.67	0.57–0.79	**<0.001**	0.66	0.56–0.78	**<0.001**
Age				1.06	1.02–1.09	**<0.001**	1.05	1.02–1.09	**0.001**
Female				1.74	1.65–1.84	**<0.001**	1.74	1.65–1.83	**<0.001**
**Education**									
Low (ref.)									
Medium				0.84	0.78–0.90	**<0.001**	0.84	0.78–0.90	**<0.001**
High				0.80	0.74–0.86	**<0.001**	0.80	0.74–0.87	**<0.001**
**Income**									
Lowest tertile (ref.)									
Middle tertile				1.03	0.96–1.09	0.409	1.02	0.96–1.09	0.511
Highest tertile				0.92	0.86–0.99	**0.017**	0.92	0.85–0.98	**0.014**
IADL				0.95	0.91–1.00	0.056	0.95	0.91–1.00	0.063
ADL				1.28	1.22–1.34	**<0.001**	1.28	1.21–1.34	**<0.001**
Chronic diseases				1.38	1.35–1.42	**<0.001**	1.38	1.34–1.42	**<0.001**
Depression (four or more depressive symptoms)				1.86	1.75–1.99	**<0.001**	1.85	1.73–1.97	**<0.001**
Affective or emotional disorders				1.52	1.35–1.72	**<0.001**	1.52	1.34–1.72	**<0.001**
Physical inactivity (yes)				1.26	1.15–1.38	**<0.001**	1.26	1.15–1.38	**<0.001**
Life satisfaction				0.84	0.82–0.87	**<0.001**	0.85	0.82–0.88	**<0.001**
Spousal care							1.30	1.13–1.48	**<0.001**
**Random parts**									
ICCcountry	0.055			0.030			0.030		
Between-country variation	0.1898			0.1021			0.1033		
Deviance	35,358.8			32,215.0			32,190.5		
N countries	17			17			17		

*Source: SHARE, release 7.0.0., wave 6, N = 2,6269. OR, Odds Ratios; CI, Confidence Intervals; p-values ICC, Intra-class Correlation Coefficients. Significant associations (p < 0.05) are bold*.

## Discussion

The current study analyzed the prevalence of pain reported by spouse caregivers aged 65+ and the association between providing spousal care and pain among European individuals aged 65 years and over. Our results are in line with previous studies showing that, in old age, spouses are an essential source of informal care (1). Our data show that 7.1% of the European population aged 65+ provide informal care to their spouse/partner inside the household. Moreover, substantial differences were found between countries regarding the prevalence of spouse caregivers among the population aged 65+, with Austria (9.9%) and Belgium (9.7%) showing the highest percentages of spouse caregivers aged 65+ and Switzerland the lowest (4%).

Concerning the prevalence of self-reported pain, our data show that more than half of our sample report pain (50.3% of non-caregivers and 60.3% of spouse caregivers). This confirms that pain is common among the older European population (24, 50). Moreover, our results also reinforce previous studies which highlight that, in later life, there is considerable variation in pain across European countries (13, 20). These results are in line with Cimas et al. (20), who found that older adults from Southern Mediterranean and Eastern countries report a higher prevalence of pain, and those living in Northern countries and Switzerland report a lower prevalence of pain.

Regarding the differences between the spouse caregiver and non-spouse caregiver groups, overall, the spouse caregiver group reported higher levels of pain compared to their non-caregiver counterparts. Moreover, significant differences between countries were also found regarding pain among spouse caregivers. While Portugal, Spain, France, Italy, and Slovenia show higher percentages of pain among spouse caregivers (above 70%), Denmark and Switzerland show significantly lower percentages (below 45%). These results reinforce the fact that spouse caregivers from Portugal, Spain, France, Italy, France, and Slovenia are confronting higher health risks. This outcome can in part be explained by the lower socio-economic position of Southern Mediterranean and Eastern countries compared with Central and North European countries (20), since people with lower socioeconomic status are more likely to experience pain (36). Moreover, the poor health of older individuals from Southern and Eastern European countries (51, 52) and the unmet long-term care needs in these countries (53) can also contribute to these findings. A recent European report (54) highlights that the level of home care resources available to older people living in their houses varies substantially within European countries, which can influence the level of care provided by informal caregivers. According to Verbakel (55), informal caregivers living in countries with strong family care norms, such as southern countries, tend to provide more intensive caregiving compared to those with generous formal long-term care provisions. Furthermore, the low levels of formal long-term care provisions can jeopardize the well-being of intensive caregivers, as well as the sustainability of the healthcare systems (55).

Therefore, considering that, in Southern and Eastern European countries, the responsibility for long-term care is mainly placed upon families (53), and the fact that there are low levels of formal long-term care provision in these countries (56), Southern and Eastern European policymakers should reassess the current social and health policies to better support informal caregivers. Greater collaboration between informal and formal care networks, as well as the implementation of adequate financial care models and the promotion of communities that integrate older citizens (54), are needed to release older spouse caregivers from their heavier care tasks.

Regarding differences between the spouse caregiver group and the non-caregiver group, our findings confirm the literature showing that individuals in the former group are older and less satisfied with their life (3) than the non-caregiver group. According to Baumann and Bucki (57) spouse caregivers with lower life satisfaction have a lifestyle that puts their health at risk. The authors found that family caregivers with low life satisfaction are more likely to feel as if they are in a permanent state of fatigue and that caregiving takes all their physical strength, which prevents caregivers from having time to relax and socialize (57).

In relation to the association between providing spousal care and pain, our analyses revealed that providing spousal care inside the household is associated with an increased likelihood of experiencing pain. Previous studies indicate that spouse caregivers have a higher likelihood of experiencing more severe effects on their health due to caregiving (4). In addition, in contrast to other types of caregiving, spouse caregivers are more likely to provide intensive care, assist with medical/nursing tasks, and are less likely to receive the help of healthcare professionals and other aides at home, (58, 59). These features can contribute to a higher risk of experiencing pain.

Considering the rapidly aging population and the fact that, in old age, spousal care is an important source of informal care, European policymakers should tailor strategies to support spouse caregivers and diminish the public health impact of pain. Moreover, since pain is associated with increased disability, healthcare utilization, and a reduction in the quality of life (24), European spouse caregivers should be supported by multidisciplinary services to prevent and alleviate their pain. In this sense, European policymakers should take measures to ensure comprehensive health and socio-economic support is provided to older European spouse caregivers.

This study has strengths and limitations. As far as we know, this is the first-ever study to analyse the association between providing spousal care at older ages and pain in several European countries. Nevertheless, this study has a number of limitations. Data from SHARE wave 6 allows us to construct the variables “reported pain,” but our literature review identifies that the majority of studies use the variable “chronic pain.” This limitation prevented us from producing a more in-depth discussion of our results and comparing them with those from other studies. In addition, our data is based on self-reported pain, which is recognized as being imprecise and subject to reporting bias (60). Moreover, the SHARE data do not indicate the number of hours of informal care provided by the spouses. We only know that this informal care is provided regularly, meaning daily or almost daily for at least 3 months. Another limitation of our study is the low number of spouse caregivers per country, which prevents us from performing a comparative analysis between countries to control for confounders. Lastly, because the current study is cross-sectional, we cannot assume causality. Future research should consider the variations in the relationship between providing spousal care and pain during people's lifetimes.

The main findings of this study are that European spouse caregivers aged 65+ report pain more often than their non-caregiver counterparts and that important differences between countries were found regarding the prevalence of pain among spouse caregivers. Moreover, after controlling for confounders, European spouse caregivers aged 65+ have a higher likelihood of reporting pain.

Considering that, in the coming years, care provided by older spouses is projected to increase, European policies should place the needs of older spousal caregivers on the political agenda. Europe, as an aging society with a higher prevalence of self-reported pain and huge disparities in terms of long-term care services, should be more aware of the pain of spouse caregivers aged 65+ as an important indicator when defining and implementing public health policies. Efforts must be made to support spouse caregivers aged 65+ by promoting health and social policies capable of preventing and reducing pain. This could improve the physical health of spouse caregivers and diminish some harmful side effects associated with pain, such as diagnosed diseases, problems with daily life activities, and a higher demand for health care (61).

## Data Availability Statement

The datasets presented in this study can be found in online repositories. The names of the repository/repositories and accession number(s) can be found below: http://www.share-project.org/data-access.html.

## Ethics Statement

SHARE project design was approved by the Ethics Council of the Max Planck Society, and by the Ethics Committees in the participating SHARE countries. The Ethics Committees waived the requirement of written informed consent for participation.

## Author Contributions

FB and AM designed the study. FB, AM, GV, and PC analyzed the data. FB wrote the manuscript. AM and PC participated in the critical review of the manuscript. All authors read and approved the final manuscript.

## Conflict of Interest

The authors declare that the research was conducted in the absence of any commercial or financial relationships that could be construed as a potential conflict of interest.
